# Modulation of antigen presenting cell functions during chronic HPV infection

**DOI:** 10.1016/j.pvr.2017.08.002

**Published:** 2017-08-18

**Authors:** Abate Assefa Bashaw, Graham R. Leggatt, Janin Chandra, Zewen K. Tuong, Ian H. Frazer

**Affiliations:** The University of Queensland Diamantina Institute, Translational Research Institute, Princess Alexandra Hospital, 37 Kent Street, Woolloongabba, Queensland 4102, Australia

## Abstract

High-risk human papillomaviruses (HR-HPV) infect basal keratinocytes, where in some individuals they evade host immune responses and persist. Persistent HR-HPV infection of the cervix causes precancerous neoplasia that can eventuate in cervical cancer. Dendritic cells (DCs) are efficient in priming/cross-priming antigen-specific T cells and generating antiviral and antitumor cytotoxic CD8+ T cells. However, HR-HPV have adopted various immunosuppressive strategies, with modulation of DC function crucial to escape from the host adaptive immune response. HPV E6 and E7 oncoproteins alter recruitment and localization of epidermal DCs, while soluble regulatory factors derived from HPV-induced hyperplastic epithelium change DC development and influence initiation of specific cellular immune responses. This review focuses on current evidence for HR-HPV manipulation of antigen presentation in dendritic cells and escape from host immunity.

## 1 Introduction

HPVs are epitheliotropic, double-stranded DNA viruses that infect basal keratinocytes (KCs) on surface epithelia of skin and mucosal membranes. Cervical and other anogenital cancers account for ~5% of the global cancer burden [1], [2] and are associated with infection of ‘high-risk’ HPVs; mainly HPV16 and HPV18. Together, HPV16 and 18 are responsible for ~70% of all cervical cancer cases worldwide, and approximately ~60% of oropharyngeal cancers are associated with HPV16 [2], [3], [4], [5], [6], [7]. More than 200 HPV genotypes have been identified. Mucosal HPVs are categorized based on their oncogenicity into high-risk (HR) and low-risk (LR) types [8], [9]. Persistence of HR-HPV infection is the key step in the transformation of normal epithelium to precancerous and cancerous lesions [10], [11]. The anogenital precancerous lesions, otherwise known as intraepithelial neoplasia, e.g. cervical intraepithelial neoplasia (CIN), can be subcategorized into low-grade (CIN1) and high-grade (CIN2/3) lesions. The development of HPV-related precancerous lesions, and cancer, is dependent on the expression of HR-HPV E6 and E7 oncoproteins; E6 and E7 oncoproteins disrupt the function of host cell cycle regulatory proteins in infected KCs and trigger cell transformation. These two oncoproteins enact cell cycle dysregulation via separate mechanisms. E6 binds to the host ubiquitin ligase E6-associated protein (E6AP/UBE3A) and promotes degradation of the p53 tumor suppressor gene product, a transcription factor promoting DNA repair, cell cycle arrest and apoptosis. In contrast, HPV E7 binds to retinoblastoma (pRb) and displaces the transcription control factor E2F, leading to constitutive expression of E2F-responsive genes, promoting cell cycle activation [12], [13], [14].

The immune system plays a key role during HPV-associated carcinogenesis. About 90% of immunocompetent HPV-infected individuals resolve a cervical infection spontaneously within three years and less than 1% develop invasive cervical cancer [15]. Cell-mediated immunity is considered to be crucial for clearance of HPV infections and HPV-related malignancy is more prevalent in immunocompromised individuals [16], [17]. The presence of a cytotoxic CD8+ T cell infiltrate in HPV-related tumors corresponds with improved patient survival [5], [18]. The entire HPV infection and life cycle of the virus is exclusively within epidermal KCs. KCs themselves are considered as a component of the innate immune system with immune sentinel functions [19], [20]. They express several toll-like receptors (TLRs) that recognize pathogen-associated molecular patterns (PAMPs) on pathogens, triggering production of type I interferon (IFN), defensins and proinflammatory cytokines such as interleukin 1 β (IL1-β) and tumor necrosis factor α (TNF-α) [21], [22].

## 2 Impact of HPV infection on KC susceptibility to immune responses

*In vitro* transfection of primary KC with episomal HPV, or HPV gene expression vectors, has demonstrated that HR-HPV gene products can prevent proinflammatory KC innate immune responses and susceptibility of KC to immune mediated elimination. Expression of the E6 and E7 genes of HR-HPV downregulates transcription and function of the viral DNA sensor, TLR9 [23], [24]. In addition, primary KCs transfected with HPV16 and HPV18 episomes show disrupted expression of inflammatory cytokine and chemokine genes [21]. HR-HPV E6/E7 oncoproteins inhibit NFκB activation and TLR-mediated proinflammatory cytokine and chemokine secretion, for proteins such as IFN-β, IL1-β, IL-8, CCL2, CCL5, and MIP3α, thus limiting innate immune cell trafficking and antigen (Ag)-specific effector cell activation. The HR-HPV oncoproteins inhibit NFκB signaling by blocking translocation of NFκB to the nucleus [25], [26], and suppressing NFκB nuclear transcriptional activities through enhancing interferon-related developmental regulator 1 (IFDR1) expression [27], and promote E6 dependent proteolytic destruction of IL-1β [28]. As a result, HPV-infected KCs fail to produce type-I IFN and the proinflammatory cytokines, TNF-α, IL-6, IL-8 and MIP3a [25], [26]. HR-HPV infection of primary human KCs also prevents IFN-γ-mediated cell-cycle arrest and blocks TNFα-mediated induction of necroptosis by downregulating interferon-induced transmembrane protein 1 (IFITM1) and receptor-interacting protein kinase 3 (RIPK3), respectively [29]. The HPV16 E5 early gene product has been shown, using immortalised HPV infected KCs, to downregulate expression of class I major histocompatibility complex (MHC-I) molecules [30], [31], reducing susceptibility of KC to CD8 T cell-mediated killing. HPV16 E7 protein expression in KCs also impairs IFN-γ-mediated enhancement of antigen (Ag) processing, presentation and cytotoxic T lymphocyte (CTL)-mediated lysis by impairing phosphorylation of STAT-1, resulting in suppression of IRF-1 mediated upregulation of TAP-1 [32], [33], [34].

## 3 Impact of HPV infection on antigen presentation to the adaptive immune system

To induce a cytotoxic T cell response against infected KCs, skin resident antigen presenting cells (APCs) must initiate and coordinate innate and adaptive immune responses. Skin resident professional APCs tissue-resident macrophages and dendritic cells (DCs). DCs are the only professional APCs that efficiently cross-present cell-associated Ags and transport processed Ag to the draining lymph nodes (LNs) to activate proliferation of naïve T cells [35], [36]. Tissue-resident-macrophages are major scavenger APCs responsible for phagocytosis of apoptotic cells and maintenance of local homeostasis. However they do not specifically migrate to the draining LNs and are less efficient in Ag presentation to T cells [36]. DCs are a highly heterogeneous group of APCs with functionally distinct subsets. Human skin DCs comprise epidermal Langerhans cells (LCs), dermal conventional DCs (cDCs) and monocyte-derived DCs, however, local inflammation is associated with a skin infiltrate of plasmacytoid DCs, the main source of type I IFN during viral infection [37], [38]. Murine models have been useful for understanding the biological functions, and dysfunctions, of antigen presenting cells in the skin under steady state and disease. It is important to note that there are fundamental differences in cellular machinery between mouse and human skin. Most studies on mouse models utilized haired skin where there is more densely packed hair follicles relative to human skin, or in the cervix, are absent. The epidermal layers of mouse and human skin are also different in thickness [39]. However, mouse skin and human skin contain similar immune cell types: Langerhans cells and tissue resident memory CD8+ T cells in the epidermis, and dermal DCs, macrophages, neutrophils, CD4+ T cells and ILCs in the dermal compartment [38]. Mouse skin also contains additional cell types such as dendritic epidermal T cells and gamma-delta T cells [40]. In healthy skin, LCs are the only resident DCs in the epidermis and account for 3–5% of epidermal nucleated cells [41], [42]. They originate from embryonic precursors before birth and, in contrast to other DC subsets, are radio-resistant. They continuously repopulate locally in the basement membrane under the epidermal layer without the need for continuous input from bone marrow precursors. Both human and murine LCs can be identified through the expression of CD11b, epithelial cell adhesion molecule (EpCAM) and langerin (CD207) [43], [44]. Dermal cDCs arise from hematopoietic precursors and mainly comprise murine CD11c+ CD11b+ and CD103+ DCs and their functionally equivalent human CD1c+ and CD141+ DCs, respectively. Mouse and human skin DCs, in both steady-state and inflammatory conditions, capture and transport cutaneous Ag to draining LNs of the T cell zone and initiate Ag-specific T cell responses [45], [46], [47]. The focus of the remainder of this review is on the influence of HPV infection on Ag presentation by professional APC in skin.

### 3.1 The effects of HPV on dendritic cell frequency and distribution

Regulating DC differentiation and function is an essential mechanism for tumor escape from immune surveillance [48]. Several studies have demonstrated that progression of HPV-induced carcinogenesis is associated with alteration of frequency and distribution of LCs, the epidermal DCs which normally reside where HPV infection occurs [49]. HR-HPV positive cancer cells, and E6- and E7-expressing cells can inhibit differentiation of monocytes into LCs *in vitro* [50]. In cervical precancerous lesions and invasive cancers, active HR-HPV infection and/or expression of E6/E7 oncoproteins were correlated with depletion of intraepithelial LCs [51], [52]. Similarly, the expression of HPV16 E7 protein alone in murine epidermis has been associated with significantly reduced numbers of epidermal LCs [53]. Besides HR-HPV-related lesions, in immunohistochemical studies of patient skin biopsies, the density of LCs was reduced in HPV8-positive *Epidermodysplasia Verruciformis* patient skin lesions [54] and in chronic HPV-associated skin warts APCs gradually disappeared from the epidermis with their retention below the basement membrane of the epidermal layer [55]. Significantly, accumulation of LCs in cervical biopsies is associated with clearance of cervical HPV infection [56]. In addition, although the number of intratumoral LC in head and neck squamous cell cancer (HNSCC) patients was higher relative to precancerous lesions, HPV-positive HNSCC patients contained a smaller number of intratumoral LCs as compared to HPV-negative HNSCC patients and increase in LC frequency has been correlated with recurrence-free survival [57]. Collectively, these data suggest that epidermal-resident LCs might be important for control of HPV-induced lesions and inhibition of LC infiltration to HPV-infected epidermis could be an immune escape mechanism of the virus.

One mechanism that could produce the altered number and distribution of LCs may be the observed downregulation in HR-HPV-infected KCs of the expression of the chemokines CCL20/MIP3α and E-cadherin [58], [59] in conjunction with inhibition of KC proinflammatory and chemotactic cytokines response [25], [26]. KCs play a significant role in the development and maintenance of epidermal LCs. KCs express CCL20 that results in migration of immature LCs to the epidermis, while E-cadherin expressed by KCs mediates adherence of LCs to KCs [60], [61], [62]. Under homeostatic conditions, IL-34 derived from KCs regulates the local replenishment of LCs [63], while KCs help maintain LCs in the epidermis via integrin-mediated activation of transforming growth factor β (TGF-β). TGF-β, an autocrine LC transcriptional factor required for its epidermal localization, is activated by integrin α_v_β_6_ and α_v_β_8_ derived from KCs [64]. Furthermore, *in vitro* experiments revealed that the KC-to-LC interaction mediated by E-cadherin is required for LC differentiation [65]. In HPV16-infected cervical epithelium, E6 and E7 expression and disease severity were correlated with downregulation of CCL20 expression [52] and HPV16 E6 and E7 silencing *in vitro* promotes CCL20 secretion and LC migration into HPV-infected epithelium [66]. Moreover, in HPV-positive HNSCC patients, immunohistochemical profiling showed that reduced CCL20 expression is correlated with a significant decrease in LC infiltration into tumors [57]. Most importantly, *in vitro* gene transfection studies in human primary KCs have demonstrated that CCL20 expression is inhibited by HPV viral proteins via downregulation of NF-κB signaling [58] and inhibition of binding of the cellular transcription factor CCAAT/enhancer binding protein (C/EBP) to a CCL20 promoter [67]. In this study, C/EBP from normal KCs colocalized with and enhanced the expression of CCL20. However, human KCs transfected with HPV8 E7 protein exhibited decreased CCL20 expression by preventing binding of C/EBP to the CCL20 promoter [67], suggesting a possible mechanism for HPV oncoprotein interference with KC-derived CCL20, which attracts LCs. Similarly, lower E-cadherin expression on HPV-infected KCs is correlated with cervical lesion severity [59], [68], [69] and reduced numbers of LCs in HPV-infected epidermis [60], [70]. *In vitro*, inhibition of HPV16 E6 and E7 oncoproteins restored E-cadherin expression and LC adhesion to KCs [69], [70]. Together, these data imply that recruitment and localization of DCs to HPV-infected epithelia may hamper accessibility of HPV proteins to APCs and this may in part contribute to failure to generate effective anti-HPV CTL responses.

### 3.2 The effects of HPV on dendritic cells activation

DC functional maturation is instrumental in generating Ag-specific immune responses. DCs continuously sample the peripheral tissues and recognize pathogens or danger-signals from dying or infected cells and undergo immunogenic maturation, characterized by transport of endosomal MHC-II to the cell membrane, up-regulation of CD80 (B7-1) and CD86 (B7-2) co-stimulatory molecules and secretion of instructive proinflammatory cytokines, including IL-1α/β, IL-6, IL-12 and TNF-α, and chemokines, including CCL2, CCL3, and CCR4 [71], [72]. Mature DCs present processed Ags in association with MHC molecules to naïve T cells, and instruct T cell proliferation by providing co-stimulatory signals and cytokines which aid the differentiation of T cells [73], [74]. However, several studies have demonstrated that HR-HPV infection leads to impaired DC maturation, thus limiting their capacity to stimulate a CTL response and this promotes lesion progression [51], [75]. T cells which encounter their cognate peptide on immature DCs that lack the co-stimulatory surface markers and/or proinflammatory cytokines are tolerized, a critical mechanism which the tumor uses to escape from host immune attacks [76].

In the context of chronic HPV infection, in addition to the low density of LCs, expression of MHC-ΙΙ and langerin on LCs in HR-HPV positive cervical low-grade CIN1 lesions was significantly reduced as compared to the normal cervical epithelium [51]. Further, in patients with HR-HPV-mediated CIN, downregulation of MHC and co-stimulatory molecules on cervical intraepithelial CD11c+ DC was significantly correlated with lesion severity [75]. An *in vitro* model using self-assembled HPV viral-like particles (VLPs) was designed to study skin-resident APC-mediated anti-HPV immune responses. *In vitro* exposure of immature LCs isolated from CIN2/3 patients [77] or healthy volunteers [78] to HPV16 L1L2 VLPs failed to up-regulate MHC and co-stimulatory molecules and did not promote secretion of proinflammatory cytokines and chemokines [77], [78]. The *in vitro* suppressive effect of VLPs is not limited to HPV16, but exposure to many HR-HPV and LR-HPV VLPs also inhibits activation of LCs [79]. In the absence of an inflammatory stimulus, CD8+ T cells co-cultured with HPV VLP exposed LCs did not proliferate or secrete the effector cytokine IFN-γ [77], [78]. On the other hand, while exposure to HPV16 L1 major capsid VLP alone triggers activation of human monocyte-derived LCs, HPV16 VLPs that contain the minor capsid protein L2, individually or with L1 were unable to induce LC activation [80]. However, human monocyte-derived DCs incubated with either HPV16 L1 or L1L2 VLPs upregulated activation molecules and were able to stimulate proliferation of T cells [80], [81]. The minor capsid protein L2-mediated interaction of HPV16 with annexin A2 heterotetramer (A2t) on LC is suggested as one possible mechanism for HPV-induced interference with LC maturation [82], leading to a failure to generate specific cellular immune responses during early HPV infection. This LC-specific inhibition by HPV L2 VLP may indicate one HPV immune escape mechanism. However, full maturation and Ag-specific CD8 T cell stimulatory capacity could be achieved when HPV VLP exposed LC were also stimulated with TLR agonists [77], [79] or cell-derived cytokine-based biologic (IRX-2) consisting of IL-1β, IL-2, IL-6, IL-8, TNF-α, GM-CSF, and IFN-γ [83], suggesting that administration of local inflammatory stimuli restore HPV capsid protein tolerized LC activation. Together, APC dysfunction during HR-HPV infection appears to be associated with accumulation of “immature” APCs, or depletion of mature DCs, in the local environment, which may consequently adversely impact on T cell priming in the draining LNs and mounting of an appropriate immune response against infected cells.

The effect of HPV-induced malignancy has been modelled in a murine skin grafting model where mice are engineered to express a HPV16 E7 transgene within basal KCs under the control of keratin 14 (K14) transcriptional promoter (K14E7). K14E7 skin displays epithelial hyperplasia that mimics HPV-related human CIN [84], [85], [86]. Similar to LCs isolated from human cervical epithelium, epidermal LCs from K14E7 transgenic mice exhibit low expression of MHC-ΙΙ and langerin/CD207, but the levels of CD40, CD80 and CD86 surface molecules were up-regulated as compared to non-transgenic mouse skin [87], [88]. Despite their partially activated state, *in vivo* Ag-processing and T cell priming capacity of K14E7 skin DCs was impaired [88]. Further, LCs in K14E7 epidermis showed a regulatory phenotype characterized by expression of indoleamine 2, 3-dioxygenase 1(IDO-1) and arginase 1 (Arg-1) [88], [89]. However, in murine epidermis transduced with HPV16 E7, depletion of LCs had no effect on suppression of CTL responses by HPV16 E7 [53]. In a murine contact hypersensitivity model, activation of skin effector T cells required antigen presentation by dermal DCs, but not LCs [90]. Therefore, whether the dysregulation of epidermal LCs is relevant to failure to initiate HPV-specific CD8+ T cell responses requires further investigation.

## 4 Chronic HPV infection, immunoregulatory cytokine milieu and DCs

### 4.1 Immunoregulatory soluble factors

**Fig. 1:**
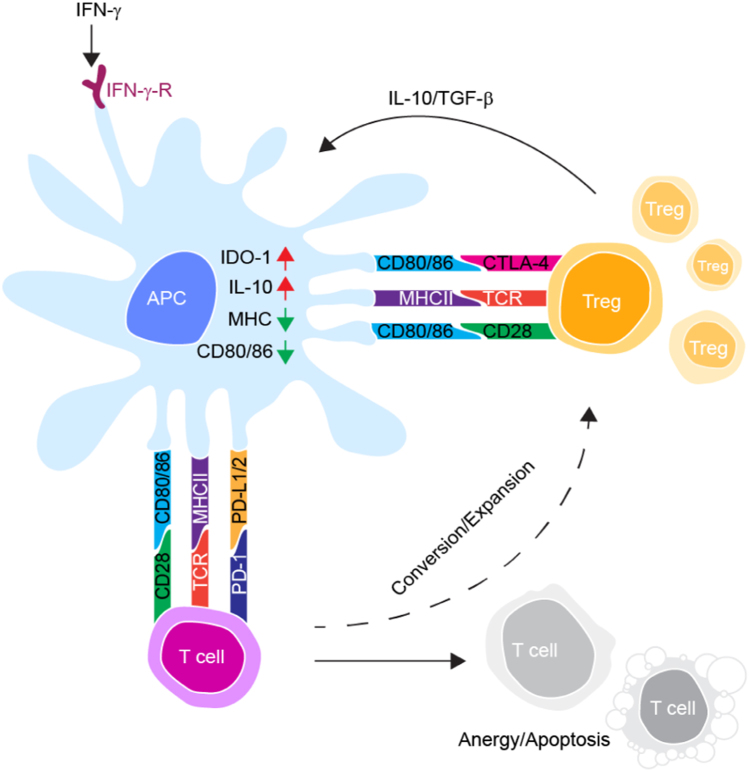
Schematic illustration of tumor -infiltrating T cell interactions with dendritic cells. Chronic IFN-γ secretion from tumor infiltrating cells and cell to cell interaction through CTLA-4 on T_reg_ cells with CD80/CD86 on DCs induce IDO expression and activities. The increased activity of IDO inhibits effector function of T cells through depleting tryptophan and increased kynurenine catabolite, kynurenine. Increase production of the immune inhibitory molecules IL-10, TGF-β and CTLA-4 in T_reg_ cells downregulate maturation of DCs. Ligation of TCR with cognate peptide-loaded MHC molecules on dendritic cells that lack the co-stimulatory surface markers and/or PD-1-PD-L1 interactions lead to T cell proliferation arrest or anergy. APC: antigen presenting cell; IDO-1: Indoleamine 2, 3-dioxygenase; IL-10: interlunkin-10; IFN-γ: Interferon γ; IFN-γ-R: IFN-γ receptor; CTLA-4: cytotoxic lymphocyte-associated antigen-4; TCR: T cell receptor; PD-1/PD-L1: programmed death receptor 1/programmed death ligand 1; TGF-β: Tumor growth factor β.

As indicated in Fig. 1 and Table 1, local immunosuppressive signaling established during precancerous epithelial neoplasia seems to play a significant role in HPV-related carcinogenesis, possibly through modulating APC activation and function. HPV-transformed cells produce immunoregulatory soluble factors and recruit suppressive cells, tumor-associated macrophages, myeloid-derived suppressor cells and regulatory T (T_reg_) cells, which instruct tolerogenic DC differentiation and promote lesion progression [91], [92]. HPV-transformed cell lines and tissues studied from patients with cervical cancer and high-grade CINs have demonstrated that the soluble mediators, IL-10, TGF-β and prostaglandins (PG), play a key role in the establishment of an immunosuppressive milieu [93], [94], [95], [96]. PGE2 produced by HPV-driven malignant epithelium inhibits DC maturation and Ag presentation [97]. In the HPV-associated neoplastic epithelium, accumulation of TGF-β and IL-10, chief regulator of DC development and function, was correlated with disease severity [92], [98]. IL-10 secreted from T_reg_ cells downregulated maturation of DCs by up-regulating the E3 ubiquitin ligase MARCH-I expression, which increased ubiquitination and degradation of MHC and costimulatory molecule [99]. The possible DC suppressive effect of IL-10 and TGF-β in a chronic HPV-infected local microenvironment is evidenced by a correlation between elevated levels of TGF-β and IL-10 positive T_reg_ cells, regulatory DCs and impaired CTL responses in cervical cancer epithelium compared to normal cervix [95]. This was also seen in mouse models where HPV Ag-experienced experimental mice produced IFN-γ, favouring the generation of IL-10+ CD4+ T cells and impaired CTL responses [100]. Additionally, *in vitro* examination of HPV-positive cervical cancer cell lines demonstrated that IL-10 and TGF-β derived from cancer cells suppress MHC-I expression in tumor cells [101], preventing CTL-mediated clearance of HPV-transformed KCs. Moreover, the expression of co-stimulatory and MHC-II in DCs co-cultured with genital squamous cell carcinoma (SCC) cell lines was significantly reduced via a receptor activator of nuclear factor kappa-B ligand (RANKL) cytokine-mediated effect although cells retained their ability to up-regulate lymphoid homing receptor CCR7, thereby inducing tolerogenic DC development [102]. The chemokine receptor CCR7 is expressed by mature DCs and plays a role in mediating DC trafficking in response to LN homing CCL19 and CCL21 chemokines [103]. Upregulated expression of CCR7 on DCs within HPV-infected squamous epithelium and with a low level of co-stimulatory markers may induce migration of immunologically inactive DCs to draining LNs [104]. In addition, HR-HPV-positive cervical cancer cell lines induced monocyte recruitment and differentiation to immature DCs *in vitro*. However, the migration of mature DCs was impaired by cervical cancer cell-derived IL-6, which led to suppression of CCR7 [105].

**Table 1:** Summary of some of the common regulatory mediators in HR-HPV- induced malignancies.

Immunoregulatory factors	Main source	Mechanisms of immune regulation	Possible effect	Reference
Arg-1	DCs and other myeloid cells	• Depletion of arginine	• Inhibit T cells proliferation and function	[88]
TGF-β & IL-10	Tumor cells, TAM, Treg cells, DCs	• Modulate DC maturation • ↓Overall immune activation	• ↑Treg cell and regulatory DC differentiation • ↓Lymphocyte functions	[91], [93], [94], [95], [96]
IFN-γ	Activated T cells	• ↑IDO and PD-L production	• ↑Treg function • ↓T cell effector function	[88], [89], [95]
PD-L1	Tumor cells, APCs	• Delivers inhibitory signal to T cells via PD1 receptor molecules	• ↓T cell activation and proliferation	[75], [93], [130]
PD-1	T cells	• Delivers intracellular negative signal	• Induce T cell anergy or exhaustion	[93], [132]
CTLA-4	T cells	• Competitively inhibit CD80 and CD86 co-stimulatory functions • Induce IDO production	• ↓T cell effector function	[107], [108], [110]
IDO-1	Tumor cells, DCs	• Depletion of tryptophan • ↑ toxic tryptophan metabolites	• ↑T cell apoptosis • ↓T cell effector function	[88], [89], [122], [123], [124]

Antigen-experienced T_reg_ cells modulate the maturation of DCs via cytotoxic T lymphocyte-antigen-4 (CTLA-4)-mediated down-regulation of CD80 and CD86 expression. CTLA-4 binds to CD80 and CD86 with higher affinity than CD28, and T_reg_ cell interaction with DCs via CTLA-4 downregulates CD80 and CD86 expression, thus limiting the ability of DCs to stimulate CD8+ T cells [106]. The expression of CTLA-4, the immune checkpoint receptor, on T_reg_ cells provides an important strategy for different tumor cells to evade host immune surveillance. Accumulation of CTLA-4+ T_reg_ cells in the HPV-positive HNSCC tumor microenvironment [107] and in the peripheral blood of advanced cervical cancer [108] patients, suggests a repressive role for CTLA-4 during HPV infection. The clinical benefits of blocking the CTLA-4 immune inhibitory pathway is also under clinical evaluation in cervical cancer [109] and HNSCC patients [110].

HPV16 E7-expressing mouse premalignant skin has also shown suppressed immune responses. Unlike skin expressing mOVA from an epidermal keratin 5 (K5) promoter- [111] or K14-driven human growth hormone (K14hGH) neo-self-antigens [112], E7-expressing murine skin is not rejected when engrafted onto immunocompetent syngeneic, non-transgenic recipient hosts [86], and an E7 immunization induced CD8+ T cell response, was not sufficient to enable graft rejection [113]. Administration of *Listeria monocytogenes* or bacterial endotoxin at the time of grafting of E7 transgenic skin promoted graft rejection [114]. This suggests that local application of proinflammatory stimuli may overcome a suppressive environment by sustaining stimulatory signals that help rescue immunogenic DC activation [115]. However, although CD4+CD25+ T cell depletion increased Ag-specific CD8 T cell responses in E7- expressing mice [116], these were insufficient to induce graft rejection [117]. Natural killer T (NKT) cells inhibit rejection of E7-grafts, and DCs isolated from NKT cell deficient mice recipient of E7 skin grafts have shown enhanced Ag-specific T cell stimulatory function in draining LNs [117]. Invariant NKT cell interaction with immature DCs can program maturation of tolerogenic DC [118] expressing immunoregulatory molecules including IDO. An increased NKT cell infiltration with increased levels of IFN-γ expression has also been observed in cervical tissues of patients with high-grade (CIN2/3) lesions [119], although the protective or suppressive nature of this response is unknown. Altogether, immune infiltrates into the tumor microenvironment may suppress anti-HPV responses by promoting regulatory DC development, thus inducing expansion of ineffective tumor infiltrating CD8+ T cells.

### 4.2 Indoleamine 2, 3-dioxygenase

Prolonged local IFN-γ-signaling contributes to E7-induced immunosuppression in K14E7 animals, as IFN-γ-deficient K14E7 skin grafts were spontaneously rejected by non-transgenic immunocompetent hosts [117]. IFN-γ exhibits anti-inflammatory and immunomodulatory functions and is the primary inducer of indoleamine 2, 3-dioxygenase (IDO) production from various cell types, including APCs and tumor cells [120]. IDO is an immunoregulatory enzyme that breaks down L-tryptophan, an important amino acid required for T cell effector function, to the immune inhibitory metabolite called kynurenine. Increased activity of IDO leads to local depletion of tryptophan and an increase in kynurenine, which subsequently inhibits the immune response by inducing apoptosis of effector T cells, converting naïve T cells into T_reg_ cells or leading to cell cycle arrest [120], [121].

In a cervical cancer patient biopsy analysis, IDO1 gene expression and kynurenine to tryptophan ratio was significantly elevated as compared to healthy controls [122]. Increased expression and activities of serum IDO [123] and cervical tumor cell IDO expression [124] were negatively associated with survival of cervical cancer patients. Markedly higher infiltration of stromal immature IDO+ DCs were observed from cervical tissues of high-grade cervical neoplastic disease and cancer [95]. Although the number of IFN-γ+ cells were lower in patients with cervical cancer compared to CIN, IFN-γ+ cells were significantly higher in high-grade CIN compared to normal cervix biopsies [95]. Indeed, IFN-γ promotes antitumor immune responses and IFN-γ producing effector T cells are important positive predictors of improved HPV-related cancer patient survival [125], [126]. However, the number of T cell populations within HPV-positive high grade CINs may not be different [119]. Further, despite the effector role of IFN-γ in antitumor immune response, there is increasing evidence that chronic IFN-γ release could promote immune suppression by upregulation of immunomodulatory signals such as PD-L1 and IDO in HPV-positive precancerous lesions [127]. Adding to the complexity of the role of IFN-γ, IFN-γ can be produced by a variety of cells, which, in combination with different tissue microenvironments, can cause different outcomes to the immune response. One source of IFN-γ is from increased numbers of invariant NKT cells, as seen in HPV-positive high-grade CIN compared to HPV-positive low-grade CINs and HPV-negative cervical tissues [119]. Invariant NKT cells and IFN-γ-dependent upregulated expression and activity of IDO-1 were detected in the K14E7 murine skin DCs and blockade of IDO-1 activities with 1-methyltryptophan (1-MT) led to rejection of E7 transgenic skin grafts [88], [89]. Additionally, in an experimental murine model of subcutaneously inoculated cervical tumor cells, though *in vitro* tumor cell growth was not affected, inhibition of IDO activity promotes significant intratumoral accumulation of natural killer (NK) cells and regression of tumor growth [128]. Collectively these data strongly suggest that prolonged or chronic IFN-γ signaling might be responsible for IDO-1 expression by HPV-infected tissue DCs and dysfunction of T cell response can be attributed to IFN-γ-induced differentiation of regulatory DCs.

### 4.3 Programmed death ligand inhibitory signaling

DCs exposed to the HR-HPV-induced tumor microenvironment may attenuate effector T cell functions through programmed death receptor 1 (PD-1)/programmed death ligand 1(PD-1/PD-L1) immune inhibitory signaling [75]. Normally, the inhibitory signaling is important to terminate cell-mediated immune responses and is responsible for preventing adverse immune reactions, such as immune-mediated destruction of normal cells during infections. The interaction between PD-1 on T cells and its ligands PD-L1 (B7-H1) and PD-L2 (B7-DC) on APCs or tumor cells delivers a negative signal that results in apoptosis and anergy or exhaustion of T cells [106]. Aberrantly high PD-1/PD-L1 signaling is associated with immune escape in cancer and HIV infection [106], [129]. K14E7 transgenic mouse skin DCs do not demonstrate elevated expression of PD-L1 [88]. However, a correlation of increased PD-1/PD-L1 expression and tumor -infiltrating T cell immune dysfunction during HR-HPV-induced carcinogenesis of both cervical [75], [93], [130] and oropharyngeal [131], [132] cancer patients has been reported. Likewise, there is significantly elevated expression of PD-1 on T cells and PD-L1 on DCs in cervical tissues [75], [93] and draining LNs [133] from patients with high-grade CIN, as compared to low-grade CIN and CIN-negative patients. Furthermore, infiltration of functionally impaired PD-1+ CD8+ T cells is observed in HNSCC [132] and cervical cancer [93] patients. In HPV-infected tissue, PD-L1-expressing DCs have shown regulatory phenotypes, characterized by reduced co-stimulatory molecule expression and IL-12 cytokine production, but up-regulated secretion of IL-10 [75]. Interaction between DCs and T cells via ligation of PD-L1/PD-1 and immunoregulatory cytokine secretion may allow for immune escape during chronic HR-HPV infection. Immune checkpoint blockade therapy can promote tumor clearance by the host immune system. *In vitro* studies involving human cell lines expressing HPV16 E7 Ags have shown that blockade of PD-1/PD-L1 signaling significantly enhanced CTL responses [93], [130]. Pre-clinical studies have also demonstrated that the combination of HPV16 E7 DNA-based therapeutic vaccines with co-blockade of PD-1/PD-L1 signaling in E6- and E7-expressing transplantable TC-1 tumor bearing mice can significantly enhance the tumor -specific CTL response and improve tumor regression [134], [135], [136]. Currently, there are several clinical trials targeting blockade of PD-1/PD-L1 in HNSCCs and nivolumab (anti-PD1 antibody) has been approved by the FDA for the treatment of recurrent or metastatic HNSCC patients [110], [137]. However, although pembrolizumab [138] and nivolumab [139] anti-PD-1 monoclonal antibodies are under phase I/II trials for the treatment of cervical and other anogenital cancers, it remains to be determined if these strategies can offer a therapeutic advantage for clearance of HPV-related tumors in patients.

## 5 Conclusion

HPV utilizes multiple strategies to evade host immune responses. HR-HPV- infected KCs escape from CTL recognition through downregulation of MHC-I molecules and the associated Ag-processing machinery. Subverting the KC’s inflammatory response by HR-HPV E6 and E7 non-structural oncoproteins contributes to the ineffectiveness of the host immune response, thus promoting persistent HPV infection and KC transformation. HPV E6 and E7 affect epidermal recruitment and localization of LCs through disrupting KC-to-LC networks. The various cell types recruited to HR-HPV infected lesions appear to interact with resident (or recruited) APCs, setting up a feed-forward loop of immunoregulation to favour persistence of infected cells. In both pre-clinical animal models and cervical cancer patients, HPV-related epithelial dysplasia is associated with infiltration of immature DCs with reduced ability to present Ags and stimulate T cell proliferation. Most strikingly, within HR-HPV-infected or E6/E7-expressing hyperproliferative epithelia, DCs exhibit regulatory phenotypes associated with production of IL-10, IDO-1, and PD-L1. Persistent immunosuppressive signaling through chronic IFN-γ, IL-10, and TGF-β in the HR-HPV-infected hyperproliferative lesion is presumably responsible for recruiting and programming tolerogenic DCs. Multiple clinical trials targeting blockade of PD-1/PD-L1 and CTLA-4 immune checkpoints are being conducted and may provide crucial help in diminishing the burden of HPV- associated diseases. However, it is not yet clear whether HR-HPV-induced malignancies directly suppress the CTL response or DCs conditioned by the regulatory milieu actively divert T cell responses towards tolerance. Thus, further investigation of the interaction between APCs and tumor -derived soluble mediators within HPV-induced malignant epithelia may help with targeting DCs for therapeutic intervention.
